# Setting the tone: crossmodal emotional face-voice combinations in continuous flash suppression

**DOI:** 10.3389/fpsyg.2024.1472489

**Published:** 2025-01-16

**Authors:** Ulrich W. D. Müller, Antje B. M. Gerdes, Georg W. Alpers

**Affiliations:** Department of Psychology, School of Social Sciences, University of Mannheim, Mannheim, Germany

**Keywords:** continuous flash suppression, crossmodality, multimodality, visual perception, threat bias, anxiety

## Abstract

Emotional stimuli are preferentially processed in the visual system, in particular, fearful faces. Evidence comes from unimodal studies with emotional faces, although real-life emotional encounters typically involve input from multiple sensory channels, such as a face paired with a voice. Therefore, in this study, we investigated how emotional voices influence preferential processing of co-occurring emotional faces. To investigate early visual processing, we used the breaking continuous flash suppression paradigm (b-CFS): We presented fearful, happy, or neutral faces to one eye, which were initially inaccessible to conscious awareness due to the predominant perception of a dynamic mask presented to the other eye. Faces were presented either unimodally or paired with non-linguistic vocalizations (fearful, happy, neutral). Thirty-six healthy participants were asked to respond as soon as the faces reached conscious awareness. We replicated earlier findings that fearful faces broke suppression faster overall, supporting a threat bias. Moreover, all faces broke suppression faster when paired with voices. Interestingly, faces paired with neutral and happy voices broke suppression the fastest, followed by faces with fearful voices. Thus, in addition to supporting a threat bias in unimodally presented fearful faces, we found evidence for crossmodal facilitation.

## Introduction

The perception of others’ emotions results from audio-visual integration of facial and vocal expressions (Collignon et al., 2008). The combined presentation of congruent faces and voices has been shown, for example, to enhance emotion recognition and to facilitate attention compared to the unimodal presentation of faces (Rigoulot and Pell, 2012; Paulmann and Pell, 2011; Palama et al., 2022; Pell et al., 2022). However, the visual processing of emotional information has been predominantly examined with isolated visual stimuli, and more research on the influences of co-occurring auditory input is needed (Gerdes et al., 2014; Schirmer and Adolphs, 2017).

An established paradigm to demonstrate preferential processing in the visual stream is Binocular Rivalry (BR) (for a review, see Blake, 2022). In the BR paradigm, two disparate stimuli are each projected to only one eye. The incompatibility of the disparate stimuli to form one coherent percept leads to the conscious perception of only one of the two stimuli at a time, with alternations between them. BR enables examining perception biases in early visual processing largely independent of intentional control and selective attention (Meng and Tong, 2004). Interestingly, systematic variations of the initially perceived stimuli and their predominance over time are not only influenced by low-level stimulus characteristics (e.g., Blake and Logothetis, 2002; Brascamp et al., 2015) but also by emotional salience (e.g., Alpers et al., 2005; Gerdes and Alpers, 2014; Müller et al., 2022). Emotional scene stimuli (positive and negative) predominate over neutral stimuli (Alpers and Pauli, 2006; Sheth and Pham, 2008). Likewise, emotional faces (happy and fearful) predominate over neutral faces, indicating preferential visual processing (e.g., Alpers and Gerdes, 2007; Bannerman et al., 2008; Quettier et al., 2021; Quettier et al., 2024; Yoon et al., 2009). However, while the effect of fearful faces seems rather robust (Hedger et al., 2016) there are conflicting findings regarding happy faces (Gayet et al., 2014).

In a variant of Binocular Rivalry (BR) called Breaking Continuous Flash Suppression (b-CFS), a stimulus is projected to one eye via a mirror stereoscope, but its perception is initially suppressed by a high-contrast stimulus continuously flashing to the other eye. This technique capitalizes on contrast and motion to induce heightened predominance in binocular rivalry (Tsuchiya and Koch, 2005). The emergence from unconscious to conscious perception of the target stimulus is thought to mirror a change of stages of visual processing (Stein and Sterzer, 2014). Therefore, the duration until the stimulus breaks into consciousness is used as an index of the strength of its preconscious visual processing (Yang and Blake, 2012). Most importantly, perceptual suppression in b-CFS is about 10 times greater than in the original BR paradigm (Tsuchiya and Koch, 2005). Therefore, b-CFS allows the presentation of visual target stimuli paired with auditory stimuli before the visual stimuli reach consciousness.

In the b-CFS variant, fearful faces, in particular, gain access to consciousness faster than neutral ones, demonstrating preferential visual processing, frequently interpreted as a threat bias (Gray et al., 2013; Oliver et al., 2015; Stein et al., 2014; Sylvers et al., 2011; Tsuchiya et al., 2009; Yang et al., 2007). However, findings in b-CFS regarding a threat bias are less consistent than in the original BR (for a review, see Hedger et al., 2016). Also, some previous studies yielded contradictory results regarding the preferential processing of happy faces in b-CFS, depending on specific low-level stimulus characteristics (see Gayet et al., 2014). In line with this, it has been argued that not the emotionality of faces per se but associated low-level differences of various facial expressions underly their preferential processing in b-CFS (e.g., possibly higher contrast in pictures with open mouth) (Gray et al., 2013; Hedger et al., 2015; for a critical review, see also Lanfranco et al., 2023a). However, even if low-level features drive the threat bias for fearful faces in b-CFS, it is important to note that this bias may still have developed for threat detection based on general-purpose sensory mechanisms (Hedger et al., 2015).

Imaging data supports the idea that visual perception in b-CFS starts preconsciously in the primary visual cortex before suppression is broken (e.g., Yamashiro et al., 2014). Regarding emotional faces, it has been suggested that the fusiform face area can be linked to non-conscious face recognition, while the amygdala and superior temporal sulcus to nonconscious facial expression recognition (e.g., Vizueta et al., 2012). However, the recognition of facial expressions outside of awareness is still being debated (for a review, see Pournaghdali and Schwartz, 2020).

Generally, research has demonstrated that sounds can modulate visual perception of neutral cues (e.g., Koelewijn et al., 2010). For example, auditory cues can entail spatial information that can modulate the detection and perception of visual targets (for reviews, see Gerdes et al., 2014; Spence, 2010). Such crossmodally enhanced activation can be found in visual cortices even for task-irrelevant sounds, and enhanced activation is associated with improved visual discrimination (Feng et al., 2014). It has also been shown that auditory cues without spatial information can modulate perception at early levels of visual processing. Specifically, detecting a visual target stimulus was enhanced by a synchronously presented abrupt tone (Vroomen and de Gelder, 2000). In addition, eye-tracking experiments found that object-specific (semantic-related) sounds facilitate the search within an early time window, including the initial saccade toward a target (Gerdes et al., 2021; Iordanescu et al., 2010).

Few studies have been conducted with multimodal stimuli in b-CFS. These demonstrate crossmodal effects of different sensory modalities on visual processing. One study showed that images, when paired with matching olfactory information, such as a rose concurrently presented with the scent of a rose, break through suppression faster in b-CFS (Zhou et al., 2010). Another b-CFS study found that when an image was superimposed with an image of a hand, it reached consciousness faster when it matched the position of the participants’ hand (Salomon et al., 2013). Regarding faces, in one study, the dynamic image of a talking face was suppressed by b-CFS and presented with a co-occurring voice speaking a sentence that either corresponded with the lip movements or not; access to awareness was facilitated when the auditory sentence matched the lip movements (Alsius and Munhall, 2013).

Concerning potential crossmodal effects on the visual processing of visual and auditory emotional material, only one recent study investigated the effect of simultaneous emotion-congruent music on the visual processing of emotional faces using the original BR paradigm (Jertberg et al., 2018). Here, rivaling positive (happy) and negative (angry, fearful) faces were paired with happy or threatening music. Negative faces were more often perceived as the initial percept, but co-occurring emotional music did not affect the initial perception of congruent emotional faces. However, emotion-congruent music fostered sustained predominance of faces, and emotion-incongruent music sustained suppression. These findings demonstrate that emotional auditory stimuli can modulate the processing of visual emotional stimuli. However, music may not be the most ecologically valid operationalization. In contrast, emotional voices are naturally linked to faces, and integrated face-voice processing at a preconscious level has been suggested (e.g., Watson et al., 2014; for reviews, see Campanella and Belin, 2007; Yovel and Belin, 2013).

Therefore, our aim in this study is twofold; first, we want to replicate earlier findings that fearful faces are processed faster in b-CFS (Hedger et al., 2016). Second, we want to investigate the visual processing of emotional faces when paired with emotional voices. Pairing emotional faces with auditory stimuli that are innately linked, such as emotional voices, may advance our understanding of crossmodal effects on visual processing.

To this end, we carried out a b-CFS experiment in which participants were presented with fearful, happy, and neutral faces in continuous flash suppression. Moreover, emotional faces were presented unimodally or paired with co-occurring fearful, happy, and neutral vocalizations. Using the b-CFS paradigm, we present emotional faces together with emotional voices before the faces reach visual consciousness. The participant’s task was to indicate by button press when faces broke suppression and reached conscious awareness.

We expected to replicate previous b-CFS studies in unimodal trials, namely a threat bias. More specifically, we hypothesize that fearful faces will break suppression faster than neutral faces, which is often seen as indication of their preferential visual processing (Gray et al., 2013; Oliver et al., 2015; Stein et al., 2014; Sylvers et al., 2011; Tsuchiya et al., 2009; Yang et al., 2007). We did not formulate a specific hypothesis regarding happy faces due to the conflicting findings in the literature (Gayet et al., 2014). Furthermore, we expected that auditory cues generally facilitate the visual processing of faces in b-CFS through crossmodally enhanced activation (e.g., Feng et al., 2014). In addition, we hypothesized that emotional vocalizations facilitate the processing of emotion-congruent faces in b-CFS, analog to emotion music in BR (Jertberg et al., 2018).

## Materials and methods

### Participants

Based on the results of a previous meta-analysis (*d* = 0.49; Hedger et al., 2016), we conducted power calculations in G*Power for (*α* = 0.05 and *β* = 0.2). Results suggested a sample size of about *N* = 35. Thirty-six participants^1^ (75% female) were tested. Due to the ongoing COVID-19 pandemic, recruitment was restricted to students and employees of the University of Mannheim and conducted under strict hygiene regulations. Most participants (86.1%) had normal or corrected-to-normal vision or only minor impairments (≤ 3 diopters). All participants reported that their vision did not hinder them from executing the tasks. The mean age of the participants was 26.72 years (range: 20–52). The participants scored in the normative range on state- (STAI-S: *M* = 37.56, *SD* = 7.86), trait- (STAI-T: *M* = 38.67, *SD* = 10.42) and social anxiety (SIAS: *M* = 15.50, *SD* = 11.46; SPS: *M* = 8.78, *SD* = 7.31). None of the participants indicated questionable data quality in the control questions (see 3.2 procedure).

### Materials and apparatus

**Figure 1:**
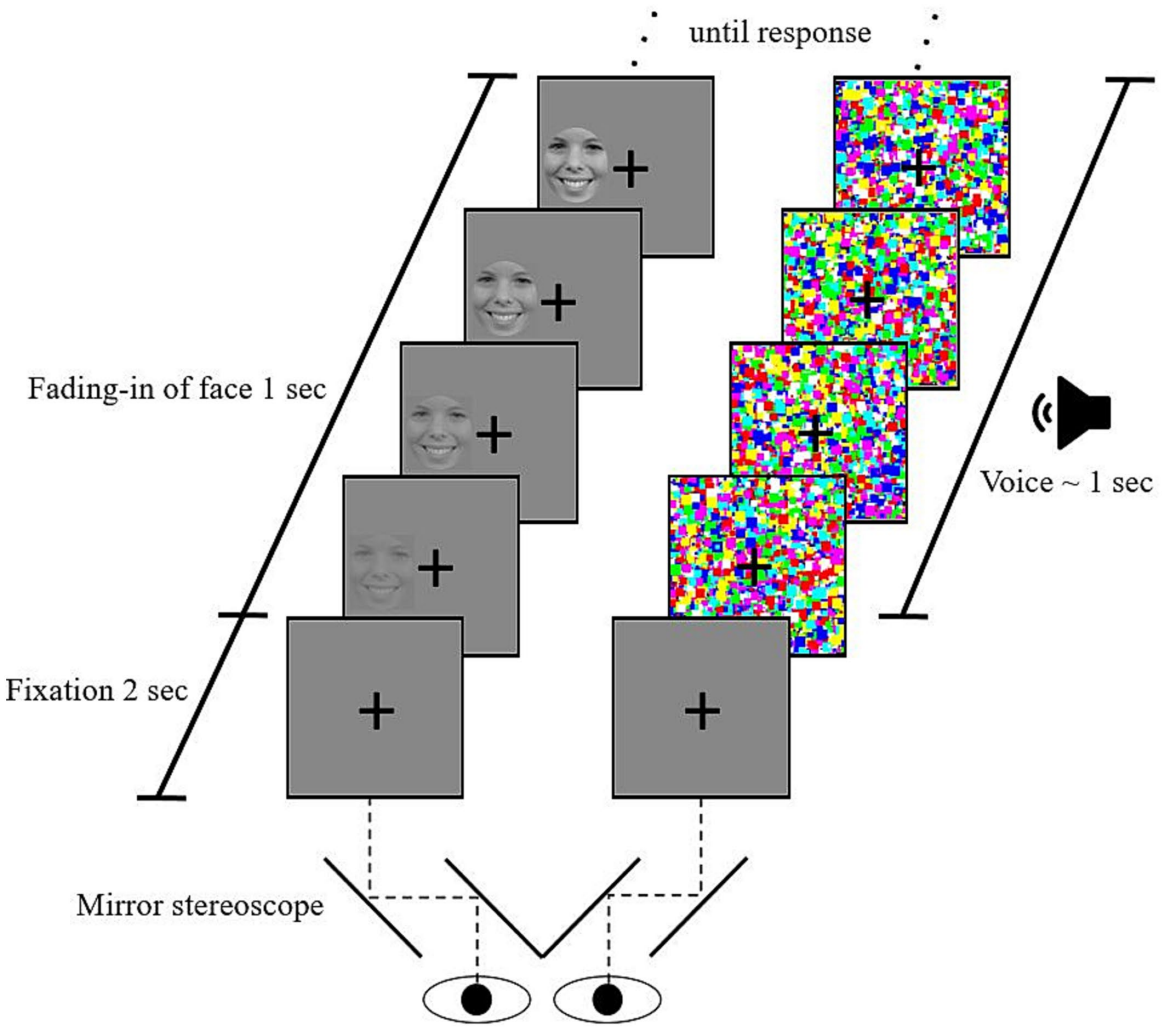
Schematic illustration of a crossmodal continuous flash suppression trial. After a 2-s fixation period in which a fixation cross is binocularly shown. The left column represents the sequence of stimulation to the left eye, the right column the flash suppression induced on the right eye (counterbalanced). An emotional voice is presented in three-quarters of the trials. Participants indicated the location of the face (left or right of the cross) by pressing the corresponding keys. A trial ended after a response was made. The bottom part illustrates how the mirror stereoscope separates the field of vision of the two eyes, thereby creating binocular rivalry between the Mondrian mask and the emotional face.

We used a b-CFS paradigm similar to previous studies (e.g., Jusyte et al., 2015; Stein et al., 2011). It was implemented in Presentation® (Version 17.2),^2^ recording participants’ responses. Each trial started with the fixation cross in a gray frame presented binocularly for 2 s. Subsequently, the dynamic mask of Mondrian-like images was presented to one eye. To the other eye, a picture of a facial expression gradually faded in on one of two possible locations (left or right of the fixation cross). Thereby, binocular rivalry between the picture and the mask was created. The face’s contrast was linearly increased, reaching full contrast after 1 s. In three-quarters of trials, an additional voice was presented via headphones simultaneous to the onset of mask and face. No voice was presented alongside the faces in one-quarter of the trials (for a schematic illustration, see Figure 1).

The b-CFS paradigm consisted of 8 practice trials to familiarize participants with the task, followed by 240 experimental trials in random order. Face emotion (fearful, happy, neutral), voice emotion (fearful, happy, neutral, no voice), portraying actress (actress 1–5), and stimulus location (left or right of fixation cross) were counterbalanced over participants so that every possible combination was presented twice - Once to the right and once to the left eye. After every 60 trials, participants could take a short break for a self-determined duration.

As picture stimuli for the experimental task, we used fearful, happy, and neutral facial expressions of five actresses (No. 1, 22, 37, 58, 61) from the Radboud Faces Database (RaFD; Langner et al., 2010). The neutral facial expressions of two further actresses from the same set (No. 12, 31) were employed for practice trials. Facial expressions in the RaFD have demonstrated high interrater reliability, with an intraclass correlation coefficient of 0.94 (Langner et al., 2010). All faces were converted to greyscale and cut out elliptically (excluding hair and background) in an attempt to minimize a priori low-level differences in luminance and contrast. For the dynamic mask, 20 colored high-contrast Mondrian-like images were continuously flashed in quick succession (10 Hz). All stimuli were presented inside grey frames with a black outline and a fixation cross in the center to facilitate binocular alignment. Auditory stimuli consisted of fearful, happy, and neutral nonverbal affect bursts of five actresses (No. 45, 46, 53, 58, 60) from the Montreal Affective Voices Database (Belin et al., 2008). The neutral voice of one actress from the same set (No. 45) was used for practice trials. A mirror stereoscope (ScreenScope SA200) with a chinrest placed 20 cm in front of a screen was used to create binocular rivalry, and voices were presented via headphones.

We administered the State–Trait Anxiety Inventory (STAI, German version; Laux et al., 1981) to assess state and trait anxiety. Social anxiety was measured by the Social Interaction Anxiety Scale (SIAS; Mattick and Clarke, 1998) and the Social Phobia Scale (SPS; Mattick and Clarke, 1998).

### Procedure

The procedure was reviewed and approved by the ethics committee of the University of Mannheim (EK Mannheim, 13/2021). After arriving at the laboratory, written informed consent was obtained from all participants. The lights were switched off during the experiment, and participants looked through the stereoscope for the b-CFS paradigm. Participants did not wear masks during the experiment, as this could hinder their ability to perform the task effectively. The experimenter requested that they remove their masks before the assessment began and then left the room.

Participants were instructed to report as soon as they saw facial features and their location by pressing specific keys on a keyboard: The right/left arrow key indicated that the facial features were perceived right/left of the fixation cross.

Afterwards, all faces and voices were presented without a stereoscope once more. Voices were presented for their predefined durations (~ 1 s) and each face was presented for 5 s. Participants were asked to rate their valence and arousal of each stimulus on a 9-point Likert scale (1 = “not at all intense”; 9 = “very intense” for arousal and 1 = “very negative”; 9 = “very positive” for valence). Subsequently, participants provided their demographics and filled in questionnaires assessing state-, trait- and social anxiety. In addition, the participants answered three control questions to ensure data quality: “Did you focus during the experiment and work thoroughly?,” “Did you answer all questions during the experiment truthfully?,” “Are there any (other) reasons not to use the data you provided?.” Finally, participants indicated whether they were familiar with the purpose of the study and were debriefed.

### Data preparation

Trials in which participants indicated wrong stimulus locations (1.76%), responses diverging more than 2 *SD* from the average response time (2.78%), and practice trials were excluded (see Capitão et al., 2014). We separately calculated mean response times in the trials for neutral, happy, and fearful faces with and without voice for each participant. Furthermore, mean response times for all face and voice combinations were calculated for each participant. After data preparation, one participant was still identified as an outlier with respect to mean response times, consistently reacting much slower than all other participants (all Face × Voice combinations >3 *SD*) and was therefore excluded from the analyses.

### Data analysis

To check if facial expressions were experienced as intended in terms of valence and arousal, we ran two separate repeated measures ANOVAs with the within-factor face emotion (neutral, happy, fearful) on the valence and arousal ratings of the faces. Likewise, to examine if the vocalizations were experienced as expected, we carried out two separate repeated measures ANOVAs with the within-factor voice emotion (neutral, happy, fearful) on the valence and arousal ratings of the voices. To follow up on significant effects, we performed t-tests between the mean ratings of the emotion categories.

To replicate previous findings and to check whether co-occurring voices facilitate the visual processing of emotional faces in b-CFS, we conducted a repeated measures ANOVA with the within-factors face emotion (neutral, happy, fearful) and voice occurrence (none, co-occurring voice) on the mean response times. We calculated planned comparisons between the face emotion categories to follow up on significant effects.

To examine the expected effects of congruency between emotional faces and emotional voices on visual processing, we conducted a repeated measures ANOVA with the within-factor face emotion (neutral, happy, fearful) and the within-factor voice emotion (neutral, happy, fearful) on the mean response times. To follow up on significant effects, we calculated planned contrasts. Confidence intervals (CI) for correlations are reported as bias-corrected bootstrap 95% intervals. In case of violation of sphericity, degrees of freedom for repeated measures were Greenhouse–Geisser corrected. *Post hoc* comparisons of individual emotion conditions were carried out for significant results only, there was no further correction for multiple comparisons to maintain statistical power of these post-hoc tests. Effect sizes for the ANOVAs are reported as partial eta squared ( $\eta_{p}^{2}$ ).

## Results

### Arousal and valence ratings of faces

In the repeated measures ANOVA for the valence ratings of the faces, we found a significant effect of face emotion [*F*_(2, 68)_ = 290.53, *p* < 0.001, $\eta_{p}^{2}$ = 0.90]. Follow-up *t*-tests showed that fearful faces (*M* = 2.70, *SD* = 1.09) were rated as more negative than happy faces (*M* = 7.30, *SD* = 0.75), *t*(34) = 18.53, *p <* 0.001, and neutral faces (*M* = 4.47, *SD* = 0.40), *t*(34) = 9.38, *p <* 0.001. Moreover, neutral faces were rated as more negative than happy faces [*t*(34) = 24.15, *p <* 0.001]. The repeated measures ANOVA for the arousal ratings of the emotional faces indicated a significant effect of face emotion [*F*_(2, 68)_ = 63.31, *p* < 0.001, $\eta_{p}^{2}$ = 0.65]. Fearful faces (*M* = 6.10, *SD* = 1.70) were rated as more arousing than happy faces (*M* = 5.40, *SD* = 1.41), *t*(34) = 2.58, *p =* 0.014, and neutral faces (*M* = 2.88, *SD* = 1.22), *t*(34) = 10.02, *p <* 0.001. Also, happy faces were rated as more arousing than neutral faces [*t*(34) = 8.14, *p <* 0.001].

### Arousal and valence ratings of voices

The repeated measures ANOVA for the valence ratings of the voices indicated a significant effect of voice emotion [*F*_(2, 34)_ = 187.98, *p* < 0.001, $\eta_{p}^{2}$ = 0.85]. Fearful voices (*M* = 2.69, *SD* = 1.17) were rated as more negative than happy voices (*M* = 7.11, *SD* = 0.96), *t*(34) = 15.03, *p <* 0.001, and neutral voices (*M* = 4.71, *SD* = 0.46), *t*(34) = 9.86, *p <* 0.001. Furthermore, neutral voices were rated as more negative than happy voices [*t*(34) = 14.42, *p <* 0.001]. In the repeated measures ANOVA for the arousal ratings of the emotional voices, we found a significant effect of voice emotion [*F*_(2, 34)_ = 98.75, *p* < 0.001, $\eta_{p}^{2}$ = 0.74]. Fearful voices (*M* = 6.22, *SD* = 1.26) were rated as more arousing than neutral voices (*M* = 2.41, *SD* = 1.33), *t*(34) = 12.44, *p <* 0.001. Moreover, happy voices (*M* = 5.69, *SD* = 1.47) were rated as more arousing than neutral voices [*t*(34) = 11.22, *p <* 0.001]. In addition, there was a trend that fearful voices were rated as more arousing than happy voices [*t*(34) = 1.88, *p =* 0.069].

### Threat bias and crossmodality

**Figure 2:**
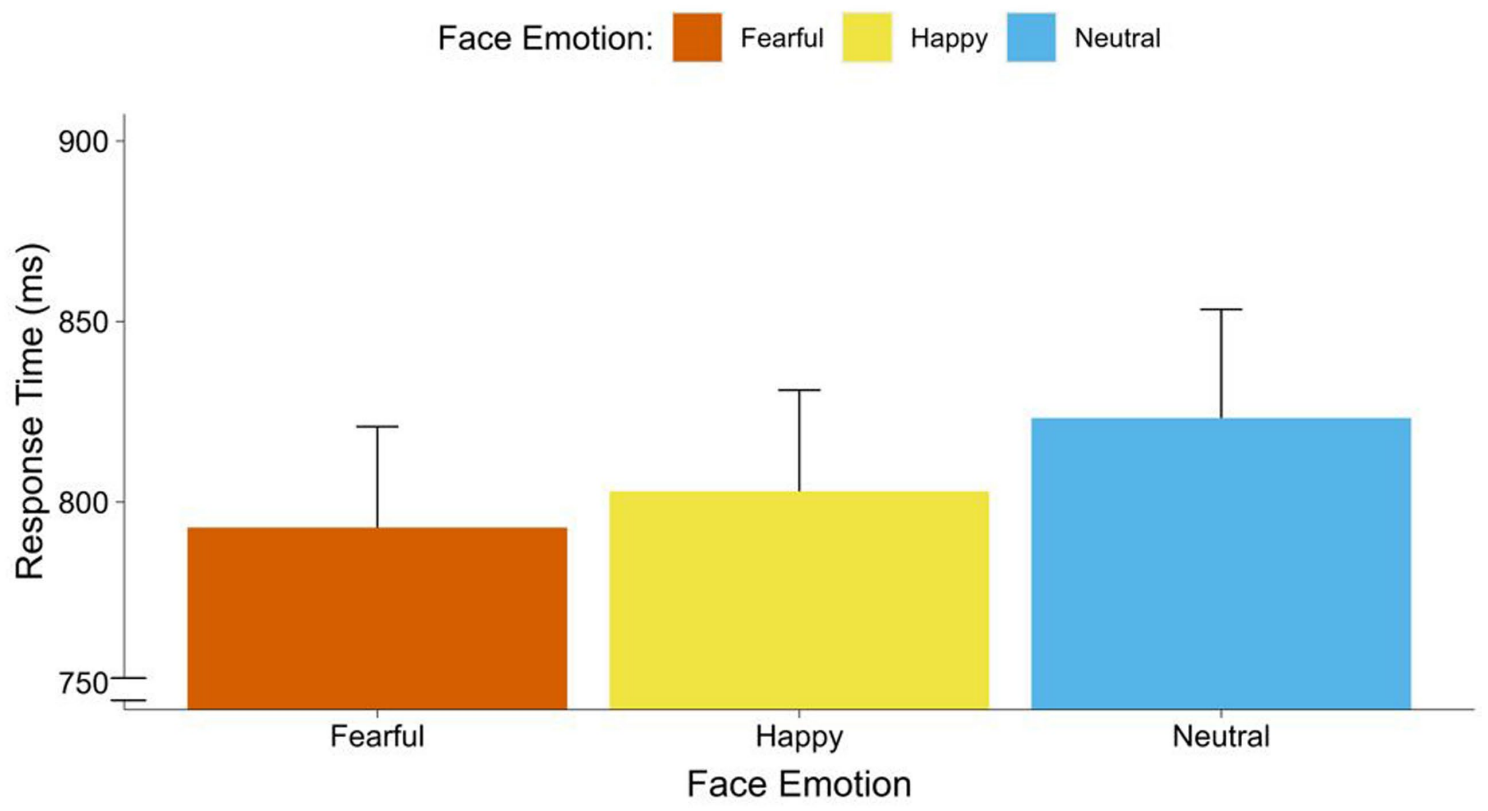
Response times of faces by face emotions. Average response times (breaking of CFS) in ms for fearful, happy, and neutral faces.

**Figure 3:**
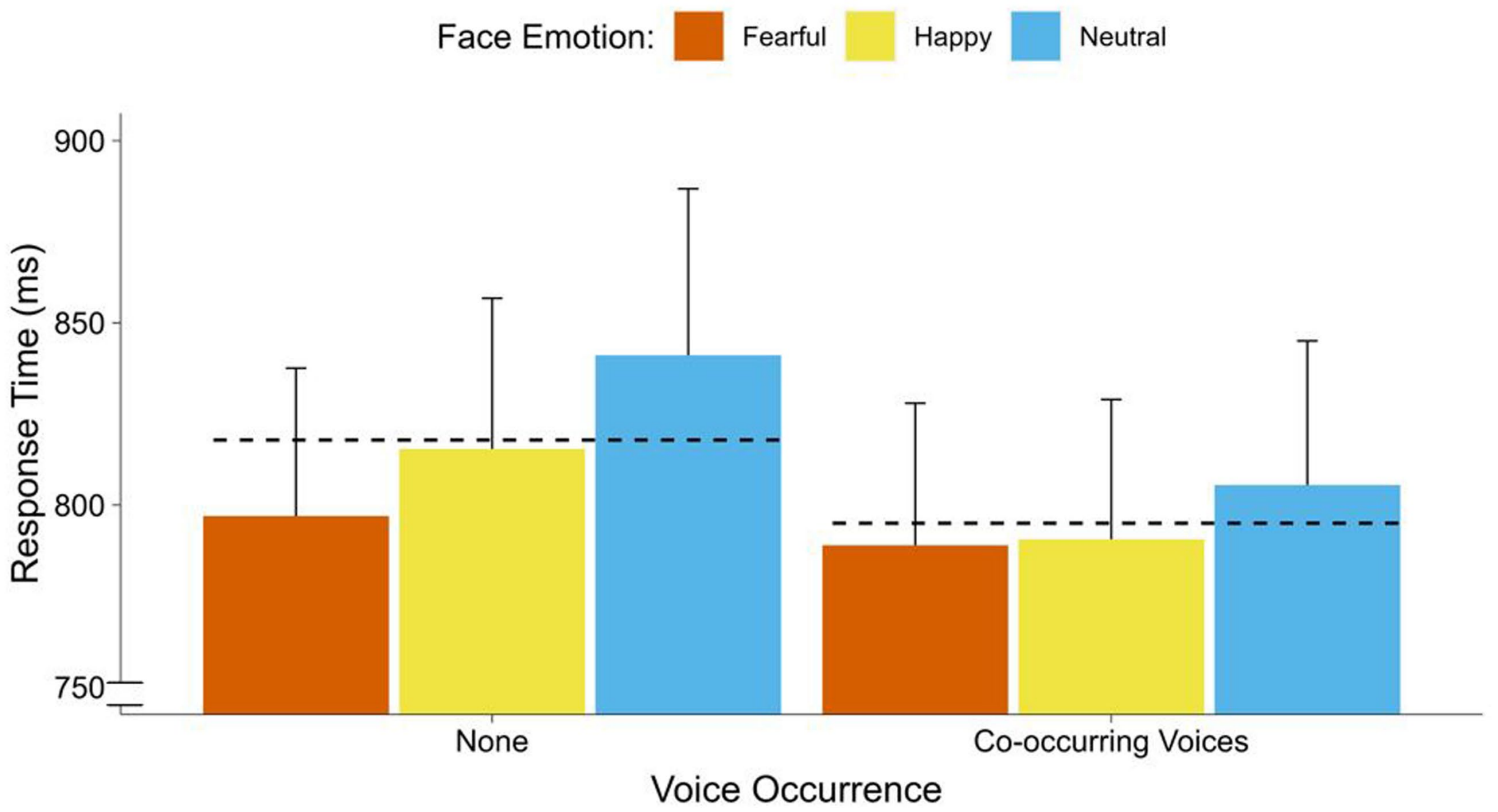
Response times of face emotions for unimodal (faces) vs. crossmodal (faces and voices) trials. Average response time (breaking of CFS) in ms for fearful, happy, and neutral faces without (three left bars) and with (three right bars) co-occurring voices. Dotted lines display the overall means for voice occurrence (none, co-occurring voice). Error bars represent standard error (SEM).

The repeated measures ANOVA with face emotion (neutral, happy, fearful) and voice occurrence (none, co-occurring voice) on response times indicated a significant main effect of face emotion [*F*_(2, 68)_ = 3.65, *p* = 0.034, $\eta_{p}^{2}$ = 0.10]. This effect was in the medium range. Contrasts showed faster responses to fearful faces [*F*_(1, 34)_ = 8.25, *p* = 0.007, $\eta_{p}^{2}$ = 0.20], than to neutral faces. Also, we found a marginally significant trend that participants responded faster to happy faces than neutral faces [*F*_(1, 34)_ = 3.49, *p* = 0.070, $\eta_{p}^{2}$ = 0.11]. There were no significant differences between fearful to faces and happy faces [*F*_(1, 34)_ = 0.12, *p* = 0.438] (see Figure 2). Moreover, there was a significant main effect of voice occurrence [*F*_(2, 34)_ = 6.05, *p* = 0.019, $\eta_{p}^{2}$ = 0.15]. This effect was in the range of a strong effect. Participants responded faster to faces with co-occurring voices than to faces with no voice. There was no significant interaction between face emotion and voice occurrence (see Figure 3).

### Differential effects of voice emotion

**Figure 4:**
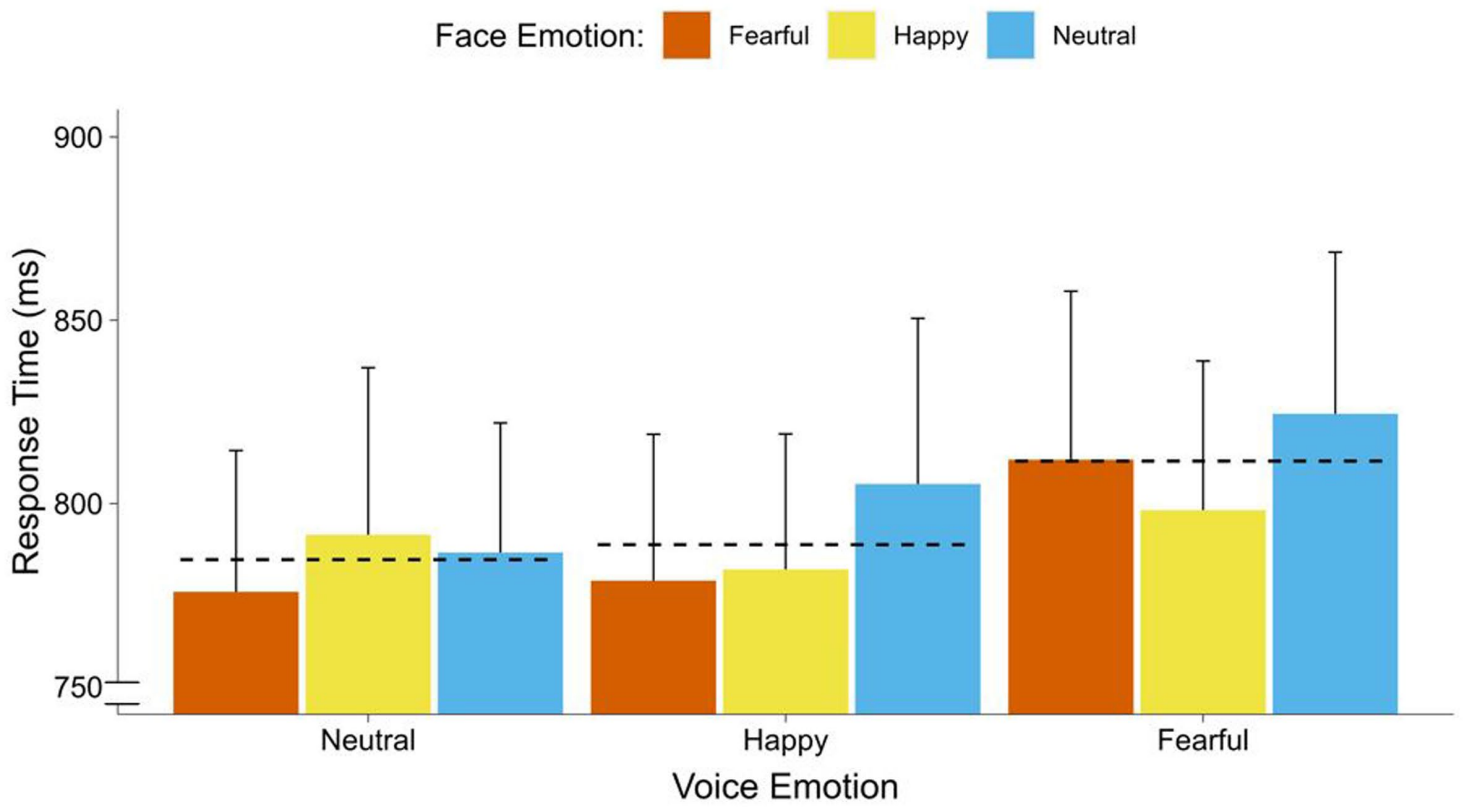
Response times of faces by co-occurring voice emotions. Average response time (breaking of CFS) in ms for faces by emotion of the co-occurring voices (fearful, happy, neutral). Dotted lines display the overall means for each voice emotion (neutral, happy, fearful). Error bars represent standard error (SEM).

In the repeated measures ANOVA, with face emotion (neutral, happy, fearful) and voice emotion (neutral, happy, fearful) on response times, we did not find a significant effect of face emotion [*F*_(2, 68)_ = 1.24, *p* = 0.296]. Furthermore, voice emotion significantly affected response times [*F*_(2, 68)_ = 5.78, *p* = 0.005, $\eta_{p}^{2}$ = 0.15] in the range of a strong effect. Contrasts indicated that participants responded faster to faces with co-occurring neutral voices [*F*_(1, 34)_ = 12.55, *p* < 0.001, $\eta_{p}^{2}$ = 0.27], and co-occurring happy voices [*F*_(1, 34)_ = 5.36, *p* = 0.027, $\eta_{p}^{2}$ = 0.14], than faces with co-occurring fearful voices. There was no difference between neutral and happy faces [*F*_(1, 34)_ = 0.27, *p* = 0.609]. Furthermore, there was no significant interaction between face emotion and voice emotion [*F*_(1, 34)_ = 0.30, *p* = 0.850] (see Figure 4).

## Discussion

Our study found that emotional faces, particularly fearful ones, gained faster access to consciousness in b-CFS. Furthermore, our findings indicate that crossmodal input affects the processing of emotional faces by modulating their access to visual consciousness. Specifically, we investigated how emotional faces are processed within the b-CFS paradigm, while also exploring potential crossmodal interactions with co-occurring emotional voices. We employed b-CFS to examine the access to consciousness of fearful, happy, and neutral facial expressions when presented either alone or paired with emotional vocalizations—neutral, happy, or fearful. Notably, this study marks the first simultaneous presentation of emotional faces and voices in the b-CFS paradigm.

Research has identified a preconscious threat bias for fearful faces across multiple paradigms (Hedger et al., 2016). Our findings further substantiate a preconscious threat bias for fearful faces in b-CFS. In addition, we found that all faces broke suppression faster when they were paired with voices. We replicated previous findings regarding a threat bias for fearful faces (Gray et al., 2013; Oliver et al., 2015; Stein et al., 2014; Sylvers et al., 2011; Tsuchiya et al., 2009; Yang et al., 2007). Specifically, in unimodal trials, fearful faces broke through suppression and became conscious faster than neutral faces. In addition, we also found a trend that happy faces were perceived faster than neutral faces.

More importantly, there is generally very limited research on the effect of co-occurring emotional information via different sensory modalities on visual processing (Gerdes et al., 2014; Schirmer and Adolphs, 2017). Moreover, unique neural mechanisms have been proposed and suggest that the integration of faces and voices starts on a preconscious level (Watson et al., 2014; for reviews, see Campanella and Belin, 2007; Yovel and Belin, 2013). B-CFS allowed us to present emotional voices simultaneously with emotional faces before the faces reached visual consciousness. As expected, participants responded faster to faces paired with voices than to faces presented alone. This suggests that co-occurring voices facilitate the visual processing of faces. This is in line with other research suggesting crossmodally enhanced activation of visual processing by co-occurring auditory cues (e.g., Feng et al., 2014). In addition, we investigated potential interactions of specific face and voice emotions. Contrary to our expectations, emotional faces paired with emotion-congruent voices did not break suppression faster. Instead, we found an effect independent of face-voice emotion congruency. Namely, co-occurring neutral and happy voices lead to faster face processing than co-occurring fearful voices. Therefore, based on our data, we cannot say whether this effect is specific to emotional voices or an instance of crossmodally enhanced visual processing by auditory input in general. This means the observed effects may reflect a more basic interaction between auditory and visual stimuli rather than a high-level emotional integration. Thus, our findings need to be interpreted with caution, as they do not support the notion of emotional congruency enhancing crossmodal processing.

Thus, our results do not align with previous research, which has suggested that congruency of crossmodal stimuli increases the strength of visual processing in b-CFS (Alsius and Munhall, 2013; Salomon et al., 2013; Zhou et al., 2010). However, none of these studies combined emotional voices with emotional faces. The study probably most similar to ours presented emotional faces alongside emotional music but in the original BR paradigm (Jertberg et al., 2018). In this study, positive (happy) and negative (angry, fearful) faces were presented in binocular rivalry and paired with happy or threatening music. It was found that congruent music did not affect the initial perception but did influence the sustained dominance of faces. Two main differences may explain why we did not find an effect of emotion congruency in our study. First, unlike emotional music, we paired emotional faces with emotional voices. Voices may differ because they rely on different specialized neural mechanisms for processing, starting preconsciously (for reviews, see Campanella and Belin, 2007; Yovel and Belin, 2013). Second, we used the b-CFS instead of the original BR paradigm. Response times in b-CFS may more closely resemble initial perception than sustained dominance in the original BR paradigm and may be better suited to detect differences in preconscious processing due to longer suppression times (Tsuchiya and Koch, 2005). This interpretation aligns with the work of Aru and Bachmann (2017), which differentiate between different phases of consciousness that are influenced by immediate iconic perception and slower memory-based experience. These phases may vary in their susceptibility to sensory input. Furthermore, recent research highlights how congruency can have distinct implications depending on the different phases during consciousness processing (Quettier et al., 2021, 2024).

Similarly, a recent study which examined both response times and neuronal responses in the context of b-CFS found generally accelerated perceptual awareness of salient visual stimuli. However, expectations or predictions regarding the following emotion category (in this case realized by written cues) did not influence the perceptual processing of emotional faces (Kalhan et al., 2022). Nevertheless, future studies should systematically investigate whether the sequence of (emotional) congruency and incongruency or specific expectations can influence visual perception during b-CFS.

One potential post-hoc explanation that faces paired with neutral or happy voices were processed faster than faces paired with fearful voices could be that fearful voices signal threat and may trigger a defensive reaction (e.g., Hamm, 2020). Such a reaction can, in turn, impair cognitive processing (Eysenck et al., 2007). In line with this reasoning, a recent study found that reactions to emotional faces paired with threat-inducing sounds were generally slower than without the sounds in an emotion recognition paradigm (Flechsenhar et al., 2022). Therefore, the longer response times to emotional faces paired with fearful voices may have been caused by the fearful voices eliciting a defensive reaction. However, it might be that such a reaction only delayed the motor responses and did not affect visual processing. Future research deploying recently developed variants of the b-CFS paradigm that allow differentiating motor activity and i.a. stimulus detection (Lanfranco et al., 2023b) or simultaneous assessment of neurological indices during b-CFS (e.g., Yuval-Greenberg and Heeger, 2013) may bring clarification.

Several limitations of this study need to be considered and should be addressed by future research. Most important, there is an ongoing and controversial debate about the extent to which accelerated response times within CFS paradigms can be interpreted as a processing advantage of emotional material on an unconscious level. For example, two comprehensive reviews (Pournaghdali and Schwartz, 2020; Lanfranco et al., 2023a) systematically investigated different experimental configurations, as well as behavioral and brain recordings in the context of CFS. Overall, the reviewed studies consistently demonstrate that low-level information such as contrast, luminance, and spatial frequency has a clear influence on conscious perception during CFS, whereas the evidence for high-level information, such as emotional salience, is less conclusive. In conclusion, the heterogeneity of experimental setups utilized by previous studies may account for conflicting evidence of the role of emotional salience in CFS.

In contrast to the positive findings already mentioned above that emotions were already processed unconsciously, there are numerous studies that report no modulation effect of emotion within CFS. Schlossmacher et al. (2017) conducted a CFS study that was combined with EEG and compared ERP components in response to emotional faces during conscious and unconscious perceptual conditions. Overall, they found no evidence for differential processing of emotional faces during continuous flash suppression whereas during the conscious condition, emotion modulated ERPs to the faces.

Likewise, a very recent study (Lanfranco et al., 2024) supports the assumption that differential emotion processing is only possible under awareness. The study measured the minimal exposure durations necessary to evoke behavioral and neural indices of (emotional) face processing, using a novel LCD tachistoscope capable of highly precise visual presentations. Results indicate a clear sequence of processing stages, requiring progressively longer exposure durations for emotion-specific processing. Thus, emotion-specific responses were only evident at durations longer than those required for reliable face detection and face-specific processing. Notably, sensitivity measures remained at chance levels until awareness was achieved.

Furthermore, existing studies showing that emotional expressions receive preferential access to awareness (see review of Lanfranco et al., 2023a) cannot effectively separate emotion detection processes from emotion identification processes. Therefore, it cannot be ruled that enhanced response times for threat stimuli may be influenced by participants’ response biases and decision-making criteria. Supporting the critical conclusions of the above-mentioned reviews, the interpretation of the threat bias in b-CFS has been also experimentally challenged recently (Lanfranco et al., 2023b). In a systematic series of experiments that employed a very high level of control in b-CFS no evidence for preferential visual processing of emotional faces was found. The authors conclude that previous findings of emotional expressions’ enhanced breakthrough into awareness is unlikely to be due to enhanced perceptual sensitivity; the source of response time acceleration is more likely to originate from one of the many other processes such as, for example physical low-level differences or different response criteria. In sum, further systematic investigations under controlled experimental conditions are needed to clarify whether emotional information can be processed without awareness within the framework of (b-)CFS. Nevertheless, rigorous control over low-level stimulus characteristics supports previous arguments that preferential processing of emotion in b-CFS is driven by associated low-level differences in facial expressions rather than emotionality per se (see Gray et al., 2013; Hedger et al., 2015; Lee et al., 2013). Fearful faces generally have higher effective contrast, caused by distinctive visual features like increased luminance contrasts around the eyes and mouth, which are thought to facilitate their breakthrough into awareness (Hedger et al., 2015). Nevertheless, this stands in contrast to research suggesting higher-order emotional processes are involved in the threat bias in b-CFS. Higher-order explanations are supported by studies showing that internal emotional states, such as anxiety (Capitão et al., 2014) and depression (Sterzer et al., 2011), can foster visual processing of stimuli displaying congruent emotions. More support comes from studies that demonstrated enhanced visual processing of neutral stimuli once associated with emotional salience (Gayet et al., 2016; Vieira et al., 2017). Importantly, even if low-level features drive the threat bias for fearful faces in b-CFS, it may still have developed for threat detection based on general-purpose sensory mechanisms (Hedger et al., 2015). While future research will need to settle this debate, studies indicating that low-level visual processing can be influenced by motivational and emotional factors (Tebbe et al., 2021) may facilitate the integration of low- and high-level explanations.

Another limitation is that our sample was a convenience sample because of the temporary restrictions due to the COVID-19 pandemic. Therefore, a few participants were not entirely naïve to the study’s purpose. However, we controlled for this and ran all analyses excluding these participants. This did not change the results in a meaningful way. Nonetheless, replications with more representative samples could reaffirm our findings. Despite using one of the few standardized and validated sets of nonverbal affective vocalizations (Belin et al., 2008), the vocalizations varied in some low-level characteristics (frequency, length). While the use of prosodic stimuli (e.g., McNally et al., 2001) would allow for better standardization of the auditory stimuli, such approach would come at the cost of external validity. Therefore, future research should investigate whether these low-level differences in auditory stimuli impact their effect on multimodal processing independent of emotional salience.

In conclusion, our study supports that a preconscious threat bias of fearful faces can be found in b-CFS. Remarkably, we also found that co-occurring auditory input facilitated the access of (emotional) faces to consciousness. Our findings indicate that crossmodal input affects the processing of emotional faces by modulating their access to visual consciousness.

## Data Availability

Data and Code will be deposited at MADATA, the data repository of the University of Mannheim and are available for future research: https://doi.org/10.7801/420.

## Footnotes

^1^As a robustness check, all analyses were also conducted excluding participants that were not fully naïve to the study’s purpose (*n* = 4). None of the here reported results changed in a meaningful way.

^2^ www.neurobs.com
